# Efficacy of Topical Beta‐Blockers in Managing Epidermal Growth Factor Receptor Inhibitor‐Related Paronychia and Pyogenic Granuloma‐Like Lesion: A Systematic Review and Meta‐Analysis

**DOI:** 10.1002/cam4.71476

**Published:** 2026-01-26

**Authors:** Po‐Kai Chan, Wei‐Ting Yen, Po‐Huang Chen

**Affiliations:** ^1^ Division of Cardiology, Department of Internal Medicine, Tri‐Service General Hospital National Defense Medical University Taipei City Taiwan (ROC); ^2^ School of Medicine, College of Medicine, National Defense Medical Universatiy Taipei City Taiwan (ROC); ^3^ Department of Ophthalmology, Tri‐Service General Hospital National Defense Medical University Taipei City Taiwan (ROC); ^4^ Division of Hematology and Oncology, Department of Internal Medicine, Tri‐Service General Hospital National Defense Medical University Taipei City Taiwan (ROC)

**Keywords:** epidermal growth factor receptor inhibitor, meta‐analysis, paronychia, pyogenic granuloma‐like lesion, topical beta‐blockers

## Abstract

**Introduction:**

Epidermal growth factor receptor (EGFR) inhibitors, including tyrosine kinase inhibitors (TKIs), are associated with paronychia and pyogenic granuloma–like lesions (PGLs) that significantly impair patients' quality of life. Topical beta‐blockers emerge as a non‐invasive and promising therapy for such adverse events. This meta‐analysis evaluated the efficacy of topical beta‐blockers for EGFR inhibitor‐induced paronychia and PGLs.

**Method:**

In accordance with the PRISMA 2020 guidelines, multiple databases were searched for relevant studies. The primary outcomes were overall response rate (ORR) and complete response rate (CRR) within 1 month. Secondary outcomes included safety outcomes and subgroup analyses, while meta‐regression was performed to assess the moderating effects of baseline characteristics. R programming was used for analysis and plotting.

**Results:**

Six studies involving 96 patients were included. Topical beta‐blocker yielded a high pooled ORR of 0.94 (confidence interval, CI 0.81–1.00, *I* ^2^ = 69%) and CRR of 0.34 (CI 0.15–0.57, *I* ^2^ = 76%) within 1 month. Despite no significant between‐group differences, lower CRR were found in lung cancer (0.35, 95% CI [0.12, 0.60]) and solution formation subgroup (0.30, 95% CI [0.06, 0.62]). Meta‐regression identified negative trends in CRRs for female patients and TKI users (*p* < 0.1). No adverse event was reported.

**Conclusion:**

Our research concluded that topical beta‐blockers may be a well‐tolerated and beneficial option for managing EGFR inhibitor‐induced paronychia and PGL. Additional and large‐scale randomized controlled trials are necessary to confirm these findings, standardize treatment protocols, and evaluate long‐term effectiveness.

## Graphical abstract

The meta‐analysis, involving with 96 patients, shows high overall response rate with favorable safety to use topical beta‐blockers for epidermal growth factor receptor inhibitors‐induced periungual side effects. Subgroups of lung cancer or solution formation had lower complete response rate, while tyrosine kinase inhibitor and female patients had negative associations.

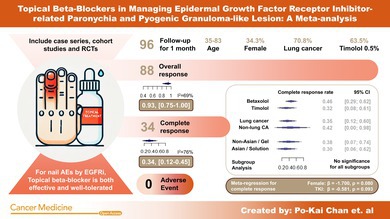

**Abbreviations**
CRR: complete response rate
EGFRi: epidermal growth factor receptor inhibitors
ORR: overall response rate
PGL: pyogenic granuloma–like lesions
TKI: tyrosine kinase inhibitors

## 1 Introduction

Epidermal growth factor receptor inhibitors (EGFRis), including anti‐EGFR monoclonal antibodies (cetuximab, panitumumab) and EGFR tyrosine kinase inhibitors (TKIs) across generations (erlotinib, gefitinib, afatinib, osimertinib), play a crucial role in treating cancers such as breast cancer, glioblastoma, gastric carcinoma, colorectal cancer (CRC), head and neck cancers, and non‐small cell lung cancer (NSCLC). However, typical side effects of EGFRis include paronychia and pyogenic granuloma–like lesions (PGLs). An early meta‐analysis estimated that periungual lesions occur in 17.2% of patients receiving EGFRis, with high‐grade lesions affecting 1.4% [1]. It has been proposed that early development and more severe EGFRi‐related skin toxicity are associated with better clinical outcomes in advanced CRC and NSCLC [2, 3]. However, these periungual adverse events impair patients' quality of life and often necessitate dose reduction or discontinuation of EGFR‐targeted therapies [4, 5], leading to poorer survival outcomes in patients who cannot continue anticancer treatment [6].

To address this issue, topical beta‐blockers have emerged as a potential treatment for EGFRi‐related paronychia and PGL, though their efficacy remains debated. Previous studies have found that β1‐ and β2‐adrenergic receptors (ARs) are expressed in skin tissues, including keratinocytes, fibroblasts, and macrophages. Topical beta‐blockers help in wound healing. Various case reports have supported the use of topical beta‐blockers for healing other difficult‐to‐treat skin conditions, including nail lesions such as post‐avulsion wounds from ingrown nails, paronychia, and PGLs [7, 8, 9, 10]. However, in one study comparing topical timolol combined with cryotherapy to cryotherapy alone, the authors concluded that adding the beta blocker did not improve clinical outcomes [11]. Thus, there is a critical need for research on the efficacy of topical beta‐blockers as a first‐line treatment to inform clinical and health policy decisions.

This study aimed to systematically review studies on the use of topical beta‐blockers in patients with EGFRi‐related paronychia and PGLs, and conduct a meta‐analysis of response rates and adverse events associated with the treatment.

## 2 Method

### 2.1 Study Design

This meta‐analysis followed the Preferred Reporting Items for Systematic Reviews and Meta‐Analyses (PRISMA) guidelines (Table S1) to assess the efficacy of topical beta‐blockers in treating EGFRi‐induced paronychia or PGLs. The primary outcomes were the overall response rate (ORR) and complete response rate (CRR), evaluated at the completion of treatment (defined as either complete lesion resolution or a maximum duration of 1 month). The secondary outcomes included safety outcomes, discontinuation of EGFRi, and subgroup analyses based on study‐level or patient‐level characteristics.

### 2.2 Search Strategy

EMBASE, Ovid‐Medline, Cochrane Central Register of Controlled Trials, ClinicalTrials.gov, and Scopus databases were searched systematically to identify relevant studies without any language restriction. PKC searched and included studies published between January 2000 and January 2025, with the last search on 10 February 2025. The search strategy involved the following keywords: “epidermal growth factor receptor inhibitors,” “paronychia,” “pyogenic granuloma,” and “topical beta‐blockers.” For a comprehensive view of the search syntax, refer to the supplement (Table S2).

### 2.3 Eligibility Criteria

Studies were included based on the following criteria (Table S3): (1) clinical trials (randomized or non‐randomized), observational studies, case series with at least five patients, (2) patients diagnosed with EGFRi‐related paronychia or PGLs, (3) treatment with topical beta‐blockers either alone or in combination with other topical agents or techniques, (4) reporting at least one clinical outcome, such as ORR, CRR, and partial response rate. Exclusion criteria included: (1) Case reports with fewer than five patients, (2) studies not using topical beta‐blockers, (3) patients without exposure to EGFRi, and (4) studies lacking detailed outcome measures related to ORR or CRR.

### 2.4 Data Extraction and Quality Assessment

Two independent reviewers (PKC and WTY) independently screened the titles and abstracts of identified articles. Full‐text articles were retrieved for studies meeting the eligibility criteria. Disagreements were resolved by consensus or consultation with a third reviewer (PHC). Data was extracted using a predesigned form on Microsoft Excel. If the study provided individual‐level characteristics or outcomes, we extracted them to assess demographic patterns and their impacts on outcomes.

### 2.5 Risk of Bias Assessment

Two reviewers (PKC and WTY) independently reviewed the quality of evidence, with discrepancies resolved by consensus. Given that topical beta‐blockers are predominantly investigated in small single‐arm trials or case series, we employed a risk of bias (RoB) assessment tool for case series [12], and the Newcastle‐Ottawa scale (NOS) for cohort studies and case series. The certainty of evidence was evaluated using a modified Grading of Recommendations Assessment, Development and Evaluation (GRADE) approach adapted for single‐arm designs. Due to the non‐comparative nature of the studies, the starting level of certainty was set to “Low.” Evidence was further downgraded based on five domains: risk of bias, inconsistency, indirectness, imprecision, and publication bias. Conversely, certainty was upgraded if a large magnitude of effect was observed.

### 2.6 Sensitivity Test, Subgroup Analysis, Meta‐Regression, and Publication Bias

Sensitivity analyses were performed to test the robustness of the findings by excluding studies with high risk of bias or extreme outcomes. A sensitivity analysis to include a longer treatment/follow‐up period (beyond 1 month) was also performed. The subgroup analyses assessed differences in outcomes based on study‐level variables. Additionally, if individual participant data and variables are available, subgroup analyses are performed based on these characteristics. Meta‐regressions were performed using a linear mixed‐effects model to assess the possible moderators. Publication bias was assessed using funnel plot analysis.

### 2.7 Data Synthesis and Statistical Analysis

Descriptive statistics of study characteristics were summarized. The pooled estimates for ORR and CRR were calculated with 95% confidence intervals (CI) using a random‐effects model with inverse variance weighting. We applied the Freeman‐Tukey double arcsine transformation in our meta‐analysis to handle extreme proportions and ensure stable variance. Heterogeneity was assessed using the *I* square (*I* ^2^) statistic and Cochran's *Q*‐test, with values of the *I* ^2^ statistic 25%, 50%, and 75% representing low, moderate, and high heterogeneity, respectively. All statistical analyses were performed using R Studio version 4.3 (RStudio: Integrated Development for R. RStudio, PBC, Boston, MA, USA). For meta‐analysis, we utilized the “meta” and “metafor” packages to pool data and generate summary estimates.

## 3 Result

### 3.1 Study Characteristics

**FIGURE 1:**
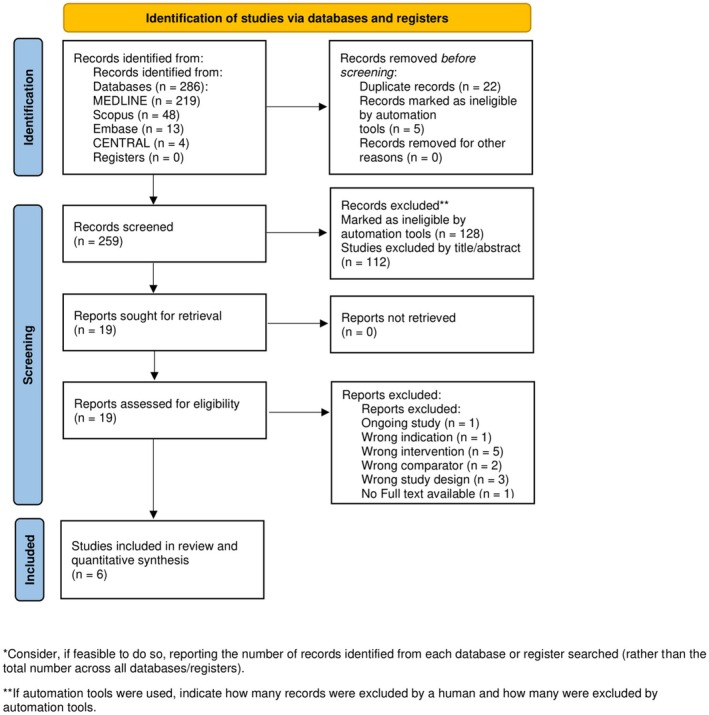
PRISMA flowchart.

The literature search process is outlined in Figure 1. 286 articles were initially identified through database searches. After screening, 19 full‐text articles were assessed, and six were included in the quantitative meta‐analysis [7, 9, 13, 14, 15, 16]. A detailed description of the search strategies, procedures, and results is provided in the supplemental material (Table S2). The six studies included 99 patients (ages 35–83), with sample sizes ranging from 9 to 35 (Table 1). Participants were primarily in their 60s and 70s, with ages ranging from 35 to 83. Five studies used timolol, while one used betaxolol. Most reported concurrent topical treatments, with few using beta‐blockers alone. One patient from Cubiro et al.'s study (on capecitabine instead of an EGFRi) and two from Yen et al.'s study (evaluated beyond 1 month) were excluded. After data cleaning, 96 patients remained for the final meta‐analysis.

**TABLE 1:** Included studies characteristics.

Study	Type of study	Cancer type	Age (range)	Cancer treatment	Topical beta‐blocker	Combined treatment	Onset, month^a^	Treatment length	Assessment timing	Patient number	Patient included^c^	Gender (female, %)	TKI, %	Lesions amount	Lesion site (H/F/B)	AEs
Cubiró, 2018, Spain	Case series	CRC, lung, H&N	60s (50s–70s)	Panitumumab Cetuximab Erlotinib Capecitabine^c^	Timolol 0.5% gel twice daily under occlusion	Not reported	3.67	1 month	1 month	10	9^c^	44.4	11.1%	27	4/4/1	Nil
Sibaud, 2019, France	Case series	NR	Median (37–80)	Lapatinib Afatinib Cetuximab Erlotinib	Timolol 0.5% gel, twice a day under occlusion (monotherapy reported)	Topical antiseptics or steroids	2.11	1 month at least	1 month	13	13	66.7	55.6%	NA	5/1/7	Nil
Sollena, 2019, Italy	Case series	CRC, lung	70.5 (65–75)	Cetuximab Afatinib Gefitinib	Timolol 0.5% gel, twice daily, under occlusion	NA	NA	Until CR or up to 4 weeks	Until CR or up to 4 weeks	9	9	61.5	76.9%	25	2/5/2	Nil
Olamiju, 2020, USA	Case series	Lung, H&N	60 (47–75)	Afatinib Osimertinib Erlotinib Cetuximab Poziotinib	Timolol 0.5% gel, twice daily, under occlusion (monotherapy reported)	Topical antiseptics or steroids, oral antibiotics, silver nitrate cauterization, cryotherapy, partial or full nail plate avulsion	6.08	4 weeks	4 weeks	10	10	50.0	71.4%	23	2/1/7	Nil
Yen, 2020, Taiwan	RCS	Lung	62 (35–83)	Afatinib Erlotinib Gefitinib	Betaxolol 0.25% solution once daily under occlusion	NA	0.50	Up to 12 weeks	Every 4 week^b^	35	35	74.3	100.0%	109	13/12/10	Nil
Liu, 2022, Taiwan	RCS	Lung	60s (50s–70s)	Afatinib Erlotinib Gefitinib Osimertinib	Timolol 0.5% solution twice daily under occlusion	Neomycin/tyrothricin ointment	7.46	9–91 days	Until treatment stop	22	20^c^	70.0	100.0%	NA	NA	Nil

Abbreviations: AEs, adverse events; CR, complete response; CRC, colorectal cancer; EGFRi, epidermal growth factor receptor inhibitor; H&N, head and neck; H/F/B, Hand/Foot/Both; NA, not analysis; NR, no response; PR, partial response; RCS, retrospective cohort studies.

^a^ Antineoplastic treatment duration before lesions developed.

^b^ In Yen et al., treatment response was assessed at 28‐day intervals, providing data for both 4‐ and 12‐week evaluations.

^c^ In Cubiró et al., 1 patient received Capecitabine instead of EGFRi, which was excluded in the final analysis. In Liu et al., 2 patients were treated and evaluated beyond 1 month, which were also removed from the final analysis.

### 3.2 Patient Characteristics

Of the 96 patients, 63 (65.6%) were female and 33 (34.3%) were male, indicating a higher proportion of female participants. Among the EGFRis used, Afatinib was the most common, accounting for 57.3% of cases, followed by Cetuximab (14.6%) and Erlotinib (11.5%). Lung cancer was the primary cancer type treated, with 68 patients (70.8%), followed by colorectal (10.4%) and head and neck cancers (6.3%). A total of 184 lesions were identified, with most people located on both hands and feet (28.1%) or on the feet alone (27.1%). The onset of lesions ranged from 1 to 39.7 months post‐EGFRi treatment (Table 2).

**TABLE 2:** Demographics of patients included in primary outcomes meta‐analysis.

Demography item	Cubiró 2018	Sibaud 2019	Sollena 2019	Olamiju 2020	Yen 2020	Liu 2022	Overall
Patient included	9	13	9	10	35	20	96
Complete response	8	2	1	4	16	3	
Partial response	1	6	7	6	19	15	
No response	0	5	1	0	0	2	
Gender^a^							
Female (*n*)	4	8	6	5	26	14	63 (65.6%)
Male (*n*)	5	5	3	5	9	6	33 (34.4%)
EGFRi^a^							
Afatinib	0	4	3	6	29	13	55 (57.3%)
Erlotinib	4	3	4	3	0	0	11 (11.5%)
Gefitinib	1	2	0	2	4	2	6 (6.3%)
Osimertinib	0	0	2	0	2	2	5 (5.2%)
Lapatinib	0	0	0	2	0	3	4 (4.2%)
Poziotinib	0	4	0	0	0	0	14 (14.6%)
Cetuximab	4	0	0	0	0	0	4 (4.2%)
Panitumumab	0	0	0	1	0	0	1 (1.0%)
Cancer type							
Colorectal	6	NR	4	0	0	0	10 (10.4%)
Lung	1	NR	5	7	35	20	68 (70.8%)
Head and neck	3	NR	0	3	0	0	6 (6.3%)
Lesion characteristics^a^							
Number of lesions	27	NR	25	23	109	32	184
Lesion/patient ratio	3.00	NA	2.78	2.30	3.11	1.60	1.92
Lesion site for each one							
Foot	4	5	2	2	13	NR	27 (28.1%)
Hand	5	1	5	1	12	NR	26 (27.1%)
Both	1	7	2	7	10	NR	24 (25.0%)
Onset (month)^b^	3.8 (1–12)	NR	2 (1–6)	4.79 (NR)	1.7 (0.5–6)	7.18 (0.82–39.02)	

Abbreviations: %, percentage; EGFRi, epidermal growth factor receptor inhibitors; *N*, number of patient; N/A, no analysis; NR, narrative report only, but no data.

^a^ Not report each patients' treatment response based on the category, thus not able to do subgroup analysis.

^b^ Antineoplastic treatment duration before lesions developed.

### 3.3 Primary Outcome

**FIGURE 2:**
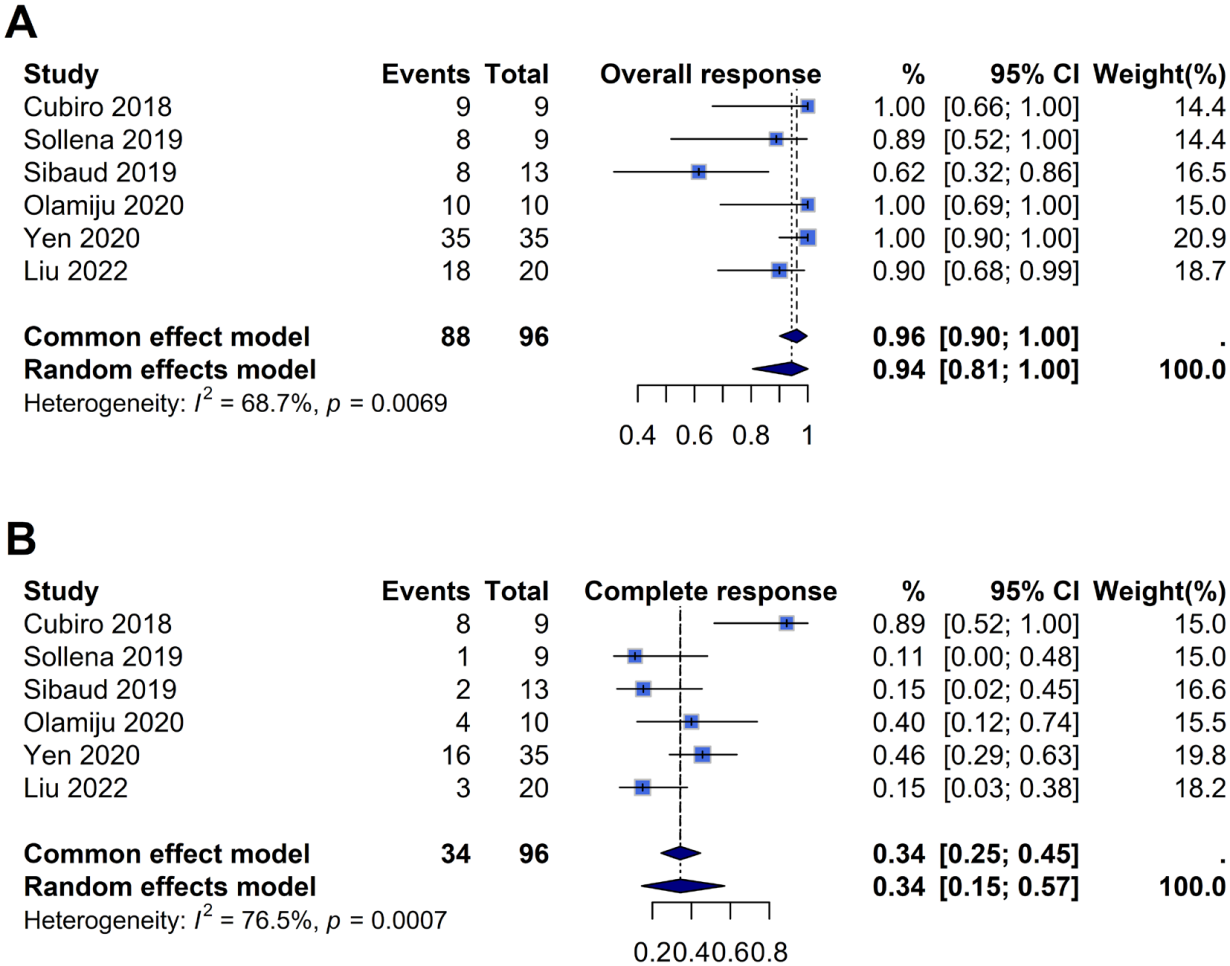
Forest plot analysis of primary outcome of (A) overall response and (B) complete response.

The meta‐analysis showed the effectiveness of topical beta‐blockers in managing EGFRi‐induced paronychia and PGL, with an ORR of 0.94 (95% CI [0.81, 1.00]) across 96 patients (Figure 2A). However, the CRR was considerably lower at 0.34 (95% CI [0.15, 0.57]), indicating that while most patients experience symptom relief or lesion improvement, full lesion resolution remains less common (Figure 2B). Overall heterogeneity was moderate for ORR (*I* ^2^ = 69%, *p* < 0.01) and higher for CRR (*I* ^2^ = 76%, p < 0.01), indicating variability across studies.

### 3.4 Secondary Outcome

#### 3.4.1 Safety and Tolerability

The safety profile of topical beta‐blockers in treating EGFRi‐related paronychia or periungual granuloma appears highly favorable, with no significant treatment‐related side effects reported (Table 1). Additionally, there were no instances of anticancer treatment discontinuation or dose modification.

#### 3.4.2 Subgroup Analysis

**FIGURE 3:**
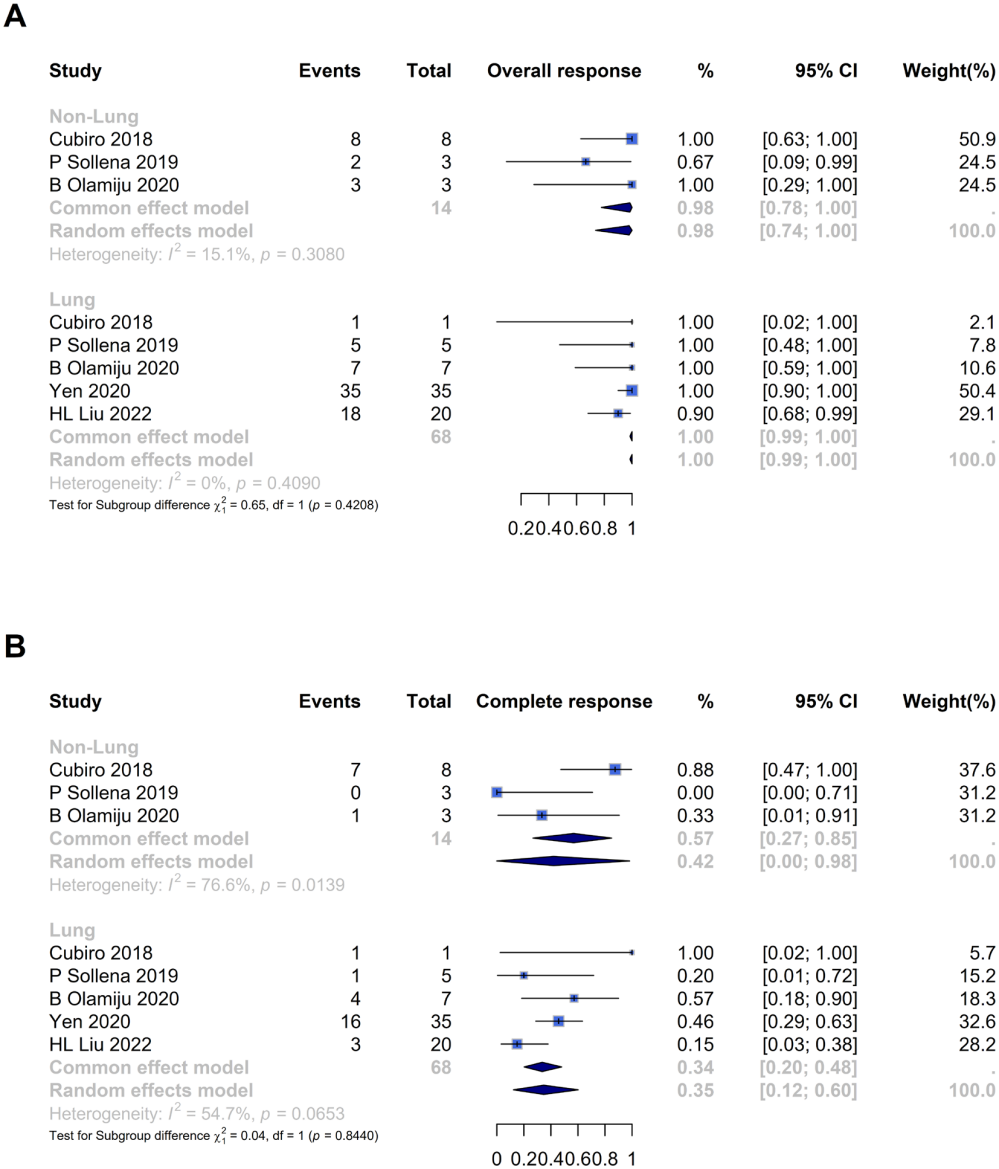
Forest plot analysis of patient‐level subgroup analysis of cancer types for (A) overall response and (B) complete response.

Five studies provided sufficient data for subgroup analysis by cancer type, shown in Figure 3. The non‐lung cancer group had a pooled ORR of 0.98 (95% CI [0.74, 1.00]) with low heterogeneity (*I* ^2^ = 15%, *p* = 0.31), while the lung cancer group showed an ORR of 1.00 (95% CI [0.99, 1.00]) with no heterogeneity (*I* ^2^ = 0%, *p* = 0.41), test for subgroup differences showed no significancy (*p* = 0.42). CRR was 0.42 (95% CI [0.00, 0.98]) in non‐lung cancer with high heterogeneity (*I* ^2^ = 77%, *p* = 0.01) and 0.35 (95% CI [0.12, 0.60]) in lung cancer with moderate heterogeneity (*I* ^2^ = 49%, *p* = 0.10), despise test for subgroup differences showed no significancy (*p* = 0.73). In terms of treatment regimens, Timolol, analyzed across five studies, had an ORR of 0.91 with moderate heterogeneity (*I* ^2^ = 54%, *p* = 0.07), while Betaxolol, evaluated only in one study, showed a 100% ORR. CRR was 0.32 for Timolol with high heterogeneity (*I* ^2^ = 79%), whereas Betaxolol had a CRR of 0.46 in its single study (Figure S1).

A geographic‐based subgroup analysis, corresponding to the use of topical gel versus solution, was also conducted. Both Asian studies from Taiwan used topical beta‐blockers in ophthalmic solutions. The non‐Asian group had an ORR of 91% (95% CI [0.69, 1.00]) with moderate heterogeneity (*I* ^2^ = 66%, *p* = 0.03), while the Asian group had an ORR of 97% (95% CI [0.81, 1.00]) with high heterogeneity (*I* ^2^ = 73%, *p* = 0.05) (Figure S2). CRR was 38% (95% CI [0.07, 0.74]) in the non‐Asian group and 30% (95% CI [0.06, 0.62]) in the Asian (or solution formulation) group, both with high heterogeneity (*I* ^2^ = 81% and 82%, respectively).

### 3.5 Meta‐Regression, Sensitivity Analysis, and Publication Bias

**FIGURE 4:**
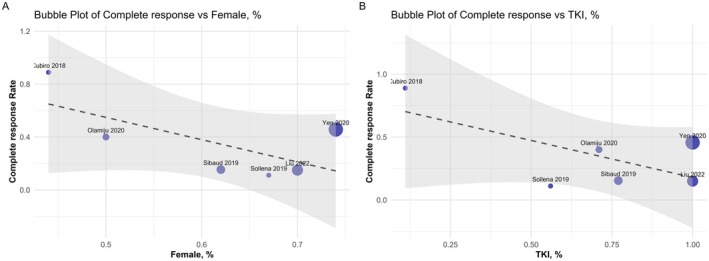
Bubble plot for meta‐regression of complete response versus (A) gender (female), and (B) TKI use.

The meta‐regression analysis results are shown in Table S5, which shows that none of the variables significantly affected CRR and ORR to topical beta‐blockers. While the *p*‐values did not reach the conventional threshold of statistical significance (*p* < 0.05), the proportion of female patients (Figure 4A) and the proportion of TKI usage (Figure 4B) appear to be negatively associated with the CRR, as both variables demonstrate a trend toward significance (*p* < 0.1).

Due to the high risk of bias in Cubiró et al., we conducted a sensitivity analysis to exclude this outlier. ORR remained high at 93% (95% CI: 0.75–1.00) with significant heterogeneity (*I* ^2^ = 74%, *p* < 0.01), while CRR was 26% (95% CI: 0.12–0.42) with moderate heterogeneity (*I* ^2^ = 55%, *p* = 0.06) (Figure S3). The reduction in CRR heterogeneity from 76% to 55% suggests that Cubiró et al.'s extreme outcomes influenced the initial analysis of lesion resolution. In the sensitivity analysis designed to ensure temporal consistency, the complete response rate rose to 47% (95% CI: 0.10–0.86) in the cohort including evaluation beyond 1 month (compared to 34% initially) with significant heterogeneity (*I* ^2^ = 70.5%, *p* < 0.01), whereas the overall response rate remained consistent at 94% (95% CI: 0.79–1.00) with significant heterogeneity (*I* ^2^ = 93.3%, *p* < 0.01) (Figure S5). The funnel plot analysis showed an asymmetric funnel (Figure S4), which may be due to small‐study effects; further larger randomized controlled trials are needed to examine the efficacy of topical beta‐blockers.

### 3.6 Risk of Bias and GRADE Assessment

Appraisal using the RoB tool developed by Murad et al. for case series and NOS suggested a moderate level of quality across the studies included in this review (Table S4). The certainty of evidence was classified as very low across all outcomes. The initial low certainty, inherent to single‐arm observational designs, was further downgraded due to serious inconsistency (indicated by high heterogeneity) and serious imprecision (attributable to small sample sizes) (Table S6).

## 4 Discussion

In this meta‐analysis, we observed a high ORR of 94% and a more modest CRR of 34% within 1 month. Notably, no adverse events were reported across the six included studies, suggesting that topical beta‐blockers are not only effective but also well‐tolerated, despite achieving less complete lesion resolution.

The therapeutic rationale for using topical beta‐blockers to manage EGFRi‐induced paronychia hinges on their capacity to counteract the cutaneous effects of EGFR blockade by modulating parallel signaling pathways. EGFR inhibitors disrupt essential cascades, such as phosphoinositide 3‐kinase/protein kinase (PI3K/Akt), Janus kinase‐signal transducer/and activator of transcription (JAK/STAT), and mitogen‐activated protein kinase/extracellular‐signal‐regulated kinase (MAPK/ERK) signaling pathways, which are critical for keratinocyte proliferation, migration, and homeostasis, leading to periungual tissue breakdown [17, 18]. In addition, EGFR inhibition induces chemokine‐mediated inflammation and compromises the periungual skin barrier, thereby increasing susceptibility to trauma and onychocryptosis that ultimately progresses to friable granulation on the lateral nail folds or distal finger nail beds, namely the formation of PGLs [19, 20].

Notably, beta‐adrenoceptors (β‐ARs) on keratinocytes modulate these signaling pathways. By acting as antagonists, topical beta‐blockers are hypothesized to prevent cytoskeletal changes, and facilitating keratinocyte migration to foster re‐epithelialization [21, 22, 23]. In addition, beta‐blockers accelerate wound healing by mitigating inflammation, promoting fibroblast migration, reducing angiogenesis inhibition, and inhibiting bacterial quorum sensing to reduce infection risk [24, 25, 26]. Through these mechanisms, topical beta‐blockers act on various stages of wound healing, supporting faster recovery and enhanced tissue repair. As in one recent review, timolol was found effective in various dermatological conditions such as epidermolysis bullosa, Kaposi sarcoma, acne rosacea, paronychia or pyogenic granulomas and infantile hemangiomas [21].

Based on the abovementioned mechanism, beta adrenoceptor antagonist was thus partially counteracting the effects of EGFRi. The high ORR observed in our meta‐analysis suggests that topical beta‐blockers are an attractive alternative with favorable safety profile and ease of administration for managing EGFRi‐induced paronychia and PGLs. Additionally, the early onset of dermatologic toxicities was reported after 4–8 weeks of use [27], which underscores the importance of early intervention to prevent progression of the lesions. In a recent clinical guideline by European Society for Medical Oncology, topical beta‐blocker had been adopted as their prevention and management of skin anticancer agents [28]. Likewise, the latest consensus by Taiwanese dermatological association and Taiwan Lung Cancer Society also suggests topical beta‐blockers as the recommended treatment on the prevention and management of EGFR TKI‐related grade 2 and 3 paronychia [29].

Furthermore, the subgroup analysis and meta‐regression provided additional insights to our results. First, despite only one study using the selective β1‐AR blocker betaxolol 0.25% ophthalmic solution, which carries a lower risk of asthma or bronchial spasm, our analysis showed a higher CRR than the pooled effects of timolol. Conducted by the same research group, betaxolol was also found effective in improving the visual analog scale for pain, severity grade [13], and even prevention for EGFR TKI‐induced paronychia [30]. A recent study found that human keratinocytes express α‐ and β‐ARs, with low epinephrine levels rapidly increasing ERK phosphorylation, suggesting α‐AR modulates β2‐AR signaling and migration [31]. Future studies could explore whether combining selective agonists and antagonists of catecholamine receptors enhances keratinocyte migration and accelerates wound healing.

Second, our study also showed there was no subgroup difference among geography difference despite lower complete response rate in the Asian subgroup. Since EGFR mutations are observed in 40%–50% of Asian populations with NSCLC compared to 10%–15% in Caucasian populations [32], TKI are widely use in this population and our analysis showed that geographic and potentially racial factors may not significantly impact the efficacy of topical beta‐blockers in treating paronychia and PGLs. However, as we state before, this subgroup analysis may also be explained by formulation differences. One previous study had argued that the timolol ophthalmic solution formulation rather than gel formulation failed in treatment of hereditary hemorrhagic telangiectasia due to inadequate tissue penetration [33]. As in our analysis, the solution formulation group had a lower complete response rate compared to the gel formulation group although not statistically significant.

Third, the Non‐Lung Cancer Subgroup Appeared to Have a Slightly Higher Complete Response Rate Compared to Lung Cancer Subgroup. Mono‐clonal EGFR antibodies, such as panitumumab and cetuximab, are more commonly used in non‐lung cancer populations and are generally associated with more severe cutaneous side effects than EGFR TKIs [20]. Consistent with this, our meta‐regression showed a minor (though non‐significant) negative trend between the proportion of TKI users and treatment response rates; this suggests that the benefits of topical beta‐blockers may be more pronounced in non‐TKI users. These findings suggest that EGFRi pharmacodynamics and pharmacokinetics may affect paronychia treatment efficacy and onset. Previous research indicated that while the high tissue penetration rates of EGFRi highlight their capacity to reach affected sites of the finger and toe, with 74.1% for erlotinib, 82.2% for gefitinib and 99.9% for afatinib, they do not necessarily correlate with paronychia severity [34, 35]. Theoretically, afatinib, with its irreversible binding to EGFR, might prolong receptor inhibition but delay symptoms resolution compared to reversible inhibitors like erlotinib and gefitinib [13]. However, the included study by Liu et al. observed a contrary outcome: 2 out of 13 patients using afatinib achieved a complete response within 1‐month treatment, whereas no patient taking Erlotinib, Osimertinib or Gefitinib experienced a complete response [16]. These findings further suggest that factors beyond drug concentration at the lesion site, such as molecular mechanisms, regional epidermal receptors expression and genetic component may impact both the intensity of cutaneous side effects. With more anticancer drugs targeting erythroblastic oncogene B and PI3K pathways, further studies should assess their impact on skin toxicity and the effectiveness of topical beta‐blockers in managing periungual side effects across different cancer types.

Our study had several limitations. First, heterogeneity in cancer types, EGFRi/TKI regimens, and beta‐blocker application protocols may introduce bias despite using a random‐effects model. Randomized trials are needed to confirm these findings and assess the long‐term safety and efficacy of topical beta‐blockers, as ongoing research investigates their role in TKI‐induced paronychia (NCT06140186) [36]. Second, small sample sizes, particularly in subgroup analyses, limit statistical power and may obscure differences between treatments; for instance, the absence of reported adverse events may reflect insufficient power to detect safety signals rather than confirming absolute safety. Lastly, future studies should also standardize adverse event assessments and report quality‐of‐life outcomes for a comprehensive evaluation. Despite these limitations, our findings align with growing evidence and provide a more robust estimate of efficacy supporting topical beta‐blockers for EGFRi‐induced dermatologic toxicities. Given the low CRR, clinicians should set realistic expectations, emphasizing symptom improvement rather than full resolution.

## 5 Conclusion

In conclusion, this meta‐analysis shows that topical beta‐blockers, may be helpful in managing EGFRi‐induced paronychia and PGLs. These findings support the use of topical beta‐blockers as a treatment option for these dermatologic toxicities. However, the modest complete response rate suggests the need for further nuanced investigation for the impact of cancer types, treatment regimens, gender differences, and variations among EGFR‐TKIs. Additional and larger randomized controlled trials are also necessary to confirm these findings, standardize treatment protocols, and evaluate long‐term effectiveness.

## Author Contributions

**Po‐Kai Chan:** conceptualization (lead), data curation (lead), formal analysis (lead), software (lead), visualization (lead), writing – original draft (lead). **Wei‐Ting Yen:** investigation (equal), methodology (equal), resources (equal), supervision (equal), validation (equal). **Po‐Huang Chen:** funding acquisition (equal), project administration (equal), resources (equal), validation (equal), writing – review and editing (equal).

## Funding

This study was supported by the Tri‐Service General Hospital (TSGH‐D‐114168, TSGH‐D‐1115176).

## Ethics Statement

This study is a meta‐analysis of previously published data and does not involve human participants. This study was exempt from ethical review by the Institutional Review Board (IRB) of the Tri‐Service General Hospital under exemption certificate number E202416043 (13 November 2024). We registered our study protocol with OSF registries (https://osf.io/d59nz) on February 17, 2025.

## Conflicts of Interest

The authors declare no conflicts of interest.

## Supporting information


**Appendix S1:** cam471476‐sup‐0001‐Supinfo.docx.

## Data Availability

The data presented in this study are available on request from the corresponding author.
