# Correction to “Efficacy of Topical Beta‐Blockers in Managing Epidermal Growth Factor Receptor Inhibitor‐Related Paronychia and Pyogenic Granuloma‐Like Lesion: A Systematic Review and Meta‐Analysis”

**DOI:** 10.1002/cam4.71647

**Published:** 2026-02-11

P. K. Chan, W. T. Yen, and P. H. Chen, “Efficacy of Topical Beta‐Blockers in Managing Epidermal Growth Factor Receptor Inhibitor‐Related Paronychia and Pyogenic Granuloma‐Like Lesion: A Systematic Review and Meta‐Analysis,” *Cancer Medicine* 15, no. 2 (2026): e71476, https://doi.org/10.1002/cam4.71476.

In the Funding section, the grant number “TSGH‐D‐1115176” was incorrect. This should have read: “TSGH‐D‐115176.” The correct Funding Statement should read: “This study was supported by the Tri‐Service General Hospital (TSGH‐D‐114168, TSGH‐D‐115176).”

We apologize for this error.

