# Correction to “Efficacy of Topical Beta‐Blockers in Managing Epidermal Growth Factor Receptor Inhibitor‐Related Paronychia and Pyogenic Granuloma‐Like Lesion: A Systematic Review and Meta‐Analysis”

**DOI:** 10.1002/cam4.72156

**Published:** 2026-07-27

P.‐K. Chan , W.‐T. Yen , and P.‐H. Chen , “Efficacy of Topical Beta‐Blockers in Managing Epidermal Growth Factor Receptor Inhibitor‐Related Paronychia and Pyogenic Granuloma‐Like Lesion: A Systematic Review and Meta‐Analysis,” Cancer Medicine 15, no. 2 (2026): e71476, 10.1002/cam4.71476.41588796 PMC12835551

The above article was originally published under the article type “Review.” The article type has been changed to “Original Research” from “Review.”

The online version of this article has been corrected accordingly.

We apologize for this error.

