# Supplementary material for: Efficacy of Topical Beta‐Blockers in Managing Epidermal Growth Factor Receptor Inhibitor‐Related Paronychia and Pyogenic Granuloma‐Like Lesion: A Systematic Review and Meta‐Analysis
Source: Cancer Med. 2026 Jan 26;15(2):e71476. doi: 10.1002/cam4.71476 (PMC12835551; doi:10.1002/cam4.71476)
Supplement: Supplementary file 1 — Appendix S1: cam471476‐sup‐0001‐Supinfo.docx. [file CAM4-15-e71476-s001.docx]

**Supplement**

**Efficacy of Topical Beta-Blockers in Managing Epidermal Growth Factor Receptor Inhibitor-Induced Paronychia and Pyogenic Granuloma-like Lesion: A Systematic Review and Meta-Analysis**

Po-Kai Chan, Wei-Ting Yen, Po-Huang Chen,

Table of contents

Table S1. PRISMA Checklist

Table S2. Search strategy

Table S3. PICO criteria

Table S4. Assessment of risk of bias

Table S5. Meta-regression results for overall response and complete response

Table S6. Summary of result and certainty of evidence

Fig S1. Forest plot analysis of study-level subgroup analysis of regimens for (A) overall response and (B) complete response

Fig S2. Forest plot analysis of study-level subgroup analysis of geographic differences for (A) overall response and (B) complete response

Fig S3: Sensitivity test of (A) overall response and (B) complete response excluding Cubiro 2018.

Fig S4. Funnel plots and Egger’s test

Fig S5. Sensitivity test of (A) overall response and (B) complete response with following period beyond 1 month.

**Table S1. PRISMA Checklist**

| **Section and Topic** | **Item #** | **Checklist item** | **Location where item is reported** |
| --- | --- | --- | --- |
| **TITLE** | | |  |
| Title | 1 | Identify the report as a systematic review. | Page 1 |
| **ABSTRACT** | | |  |
| Abstract | 2 | See the PRISMA 2020 for Abstracts checklist. | Page 8 |
| **INTRODUCTION** | | |  |
| Rationale | 3 | Describe the rationale for the review in the context of existing knowledge. | Page 10-11 |
| Objectives | 4 | Provide an explicit statement of the objective(s) or question(s) the review addresses. | Page 11 |
| **METHODS** | | |  |
| Eligibility criteria | 5 | Specify the inclusion and exclusion criteria for the review and how studies were grouped for the syntheses. | Page 12 |
| Information sources | 6 | Specify all databases, registers, websites, organisations, reference lists and other sources searched or consulted to identify studies. Specify the date when each source was last searched or consulted. | Page 11 |
| Search strategy | 7 | Present the full search strategies for all databases, registers and websites, including any filters and limits used. | Supplement S1-2 |
| Selection process | 8 | Specify the methods used to decide whether a study met the inclusion criteria of the review, including how many reviewers screened each record and each report retrieved, whether they worked independently, and if applicable, details of automation tools used in the process. | Page 12 |
| Data collection process | 9 | Specify the methods used to collect data from reports, including how many reviewers collected data from each report, whether they worked independently, any processes for obtaining or confirming data from study investigators, and if applicable, details of automation tools used in the process. | Page 12 |
| Data items | 10a | List and define all outcomes for which data were sought. Specify whether all results that were compatible with each outcome domain in each study were sought (e.g. for all measures, time points, analyses), and if not, the methods used to decide which results to collect. | Page 11 |
|  | 10b | List and define all other variables for which data were sought (e.g. participant and intervention characteristics, funding sources). Describe any assumptions made about any missing or unclear information. | Page 12 |
| Study risk of bias assessment | 11 | Specify the methods used to assess risk of bias in the included studies, including details of the tool(s) used, how many reviewers assessed each study and whether they worked independently, and if applicable, details of automation tools used in the process. | Page 12 |
| Effect measures | 12 | Specify for each outcome the effect measure(s) (e.g. risk ratio, mean difference) used in the synthesis or presentation of results. | Page 13 |

| Synthesis methods | 13a | Describe the processes used to decide which studies were eligible for each synthesis (e.g. tabulating the study intervention characteristics and comparing against the planned groups for each synthesis (item #5)). | Page 12 |
| --- | --- | --- | --- |
|  | 13b | Describe any methods required to prepare the data for presentation or synthesis, such as handling of missing summary statistics, or data conversions. | Page 13 |
|  | 13c | Describe any methods used to tabulate or visually display results of individual studies and syntheses. | Page 13 |
|  | 13d | Describe any methods used to synthesize results and provide a rationale for the choice(s). If meta-analysis was performed, describe the model(s), method(s) to identify the presence and extent of statistical heterogeneity, and software package(s) used. | Page 13 |
|  | 13e | Describe any methods used to explore possible causes of heterogeneity among study results (e.g. subgroup analysis, meta-regression). | Page 13 |
|  | 13f | Describe any sensitivity analyses conducted to assess robustness of the synthesized results. | Page 13 |
| Reporting bias assessment | 14 | Describe any methods used to assess risk of bias due to missing results in a synthesis (arising from reporting biases). | - |
| Certainty assessment | 15 | Describe any methods used to assess certainty (or confidence) in the body of evidence for an outcome. | - |
| **RESULTS** | | |  |
| Study selection | 16a | Describe the results of the search and selection process, from the number of records identified in the search to the number of studies included in the review, ideally using a flow diagram. | Figure 1 |
|  | 16b | Cite studies that might appear to meet the inclusion criteria, but which were excluded, and explain why they were excluded. | - |
| Study characteristics | 17 | Cite each included study and present its characteristics. | Table 1 |
| Risk of bias in studies | 18 | Present assessments of risk of bias for each included study. | Supplement S4 |
| Results of individual studies | 19 | For all outcomes, present, for each study: (a) summary statistics for each group (where appropriate) and (b) an effect estimate and its precision (e.g. confidence/credible interval), ideally using structured tables or plots. | Table 1 & 2,  Figure 1  Main text p14 |
| Results of syntheses | 20a | For each synthesis, briefly summarise the characteristics and risk of bias among contributing studies. |  |
|  | 20b | Present results of all statistical syntheses conducted. If meta-analysis was done, present for each the summary estimate and its precision (e.g. confidence/credible interval) and measures of statistical heterogeneity. If comparing groups, describe the direction of the effect. | Page 15-16 |
|  | 20c | Present results of all investigations of possible causes of heterogeneity among study results. | Page 15 |
|  | 20d | Present results of all sensitivity analyses conducted to assess the robustness of the synthesized results. | Page 16 |
| Reporting biases | 21 | Present assessments of risk of bias due to missing results (arising from reporting biases) for each synthesis assessed. | Page 16 |
| Certainty of evidence | 22 | Present assessments of certainty (or confidence) in the body of evidence for each outcome assessed. | - |
| **DISCUSSION** | | |  |
| Discussion | 23a | Provide a general interpretation of the results in the context of other evidence. | Page 17-21 |
|  | 23b | Discuss any limitations of the evidence included in the review. | Page 21 |
|  | 23c | Discuss any limitations of the review processes used. | Page 21 |
|  | 23d | Discuss implications of the results for practice, policy, and future research. | Page 22 |
| **OTHER INFORMATION** | | |  |
| Registration and protocol | 24a | Provide registration information for the review, including register name and registration number, or state that the review was not registered. | Page 3 |
|  | 24b | Indicate where the review protocol can be accessed, or state that a protocol was not prepared. | Page 3 |
|  | 24c | Describe and explain any amendments to information provided at registration or in the protocol. | Page 3 |
| Support | 25 | Describe sources of financial or non-financial support for the review, and the role of the funders or sponsors in the review. | Page 3 |
| Competing interests | 26 | Declare any competing interests of review authors. | Page 3 |
| Availability of data, code and other materials | 27 | Report which of the following are publicly available and where they can be found: template data collection forms; data extracted from included studies; data used for all analyses; analytic code; any other materials used in the review. | Page 3 |

*From:*  Page MJ, McKenzie JE, Bossuyt PM, Boutron I, Hoffmann TC, Mulrow CD, et al. The PRISMA 2020 statement: an updated guideline for reporting systematic reviews. BMJ 2021;372:n71. doi: 10.1136/bmj.n71

**Table S2. Search strategy**

| **Pubmed** |
| --- |
| ("paronychia"[MeSH Terms] OR "paronychia"[All Fields] OR "paronychias"[All Fields] OR (("pyogen"[All Fields] OR "pyogenic"[All Fields] OR "pyogenous"[All Fields]) AND "granuloma-like"[All Fields] AND ("lesion"[All Fields] OR "lesion s"[All Fields] OR "lesional"[All Fields] OR "lesions"[All Fields])) OR ("granuloma, pyogenic"[MeSH Terms] OR ("granuloma"[All Fields] AND "pyogenic"[All Fields]) OR "pyogenic granuloma"[All Fields] OR ("pyogenic"[All Fields] AND "granuloma"[All Fields]))) AND ((("erbb receptors"[MeSH Terms] OR ("erbb"[All Fields] AND "receptors"[All Fields]) OR "erbb receptors"[All Fields] OR "egfr"[All Fields]) AND ("antagonists and inhibitors"[MeSH Subheading] OR ("antagonists"[All Fields] AND "inhibitors"[All Fields]) OR "antagonists and inhibitors"[All Fields] OR "inhibitors"[All Fields] OR "inhibitor"[All Fields] OR "inhibitor s"[All Fields])) OR (("erbb receptors"[MeSH Terms] OR ("erbb"[All Fields] AND "receptors"[All Fields]) OR "erbb receptors"[All Fields] OR ("epidermal"[All Fields] AND "growth"[All Fields] AND "factor"[All Fields] AND "receptor"[All Fields]) OR "epidermal growth factor receptor"[All Fields]) AND ("antagonists and inhibitors"[MeSH Subheading] OR ("antagonists"[All Fields] AND "inhibitors"[All Fields]) OR "antagonists and inhibitors"[All Fields] OR "inhibitors"[All Fields] OR "inhibitor"[All Fields] OR "inhibitor s"[All Fields]))) AND ((("topical"[All Fields] OR "topically"[All Fields] OR "topicals"[All Fields]) AND ("adrenergic beta antagonists"[Pharmacological Action] OR "adrenergic beta antagonists"[MeSH Terms] OR ("adrenergic"[All Fields] AND "beta antagonists"[All Fields]) OR "adrenergic beta antagonists"[All Fields] OR ("beta"[All Fields] AND "blocker"[All Fields]) OR "beta blocker"[All Fields])) OR ("timolol"[MeSH Terms] OR "timolol"[All Fields])) |
| **Embase** |
| ('protein tyrosine kinase inhibitor'/exp OR 'epidermal growth factor receptor'/exp OR 'erbb 1 protein' OR 'erbb 1 receptor' OR 'erbb1 protein' OR 'erbb1 receptor' OR 'egf receptor' OR 'epidermal growth factor receptor' OR 'epidermal growth factor receptor 1' OR 'epidermal growth factor receptor kinase' OR 'epidermal growth factor receptor protein tyrosine kinase' OR 'epidermal growth factor receptor protein-tyrosine kinase' OR 'epidermal growth factor receptor tyrosine kinase' OR 'epidermis growth factor receptor' OR 'epidermis growth factor receptor kinase' OR 'erbb receptor' OR 'erbb receptors' OR 'protein erbb' OR 'protein erbb 1' OR 'protein erbb1' OR 'receptor, epidermal growth factor' OR 'receptors, epidermal growth factor-urogastrone' OR 'transforming growth factor alpha receptor') AND ('paronychia'/exp OR 'panaris' OR 'paronychia' OR 'paronychium' OR 'perionychia' OR 'pyogenic granuloma'/exp OR 'angiogranuloma' OR 'granulation tissue haemangioma' OR 'granulation tissue hemangioma' OR 'granulation tissue-type haemangioma' OR 'granulation tissue-type hemangioma' OR 'granuloma gravidarum' OR 'granuloma pyogenicum' OR 'granuloma pyogenicum telangiectaticum' OR 'granuloma pyogenicum teleangiectaticum' OR 'granuloma pyogenicum teleangiectatum' OR 'granuloma telangiectaticum' OR 'granuloma teleangiectaticum' OR 'granuloma, pyogenic' OR 'granulomata pyogenicum' OR 'haemangioma, granulation tissue' OR 'hemangioma, granulation tissue' OR 'pyogenic granuloma') AND ('beta adrenergic receptor blocking agent'/exp OR 'timolol'/exp OR '1 (tert butylamino) 3 (4 morpholino 1, 2, 5 thiadiazol 3 yloxy) 2 propanol' OR '1 tert butylamino 3 (4 morpholino 1, 2, 5 thiadiazol 3 yloxy) 2 propanol' OR '1 tert butylamino 3 (4 morpholino 1, 2, 5 thiadiazol 3 yloxy) propan 2 ol' OR '3 (3 tert butylamino 2 hydroxypropoxy) 4 morpholino 1, 2, 5 thiadiazole' OR 'apo timolol' OR 'apo-timolol' OR 'apotimolol' OR 'l 714465' OR 'l714465' OR 'timolo' OR 'timolol' OR 'timolol hemihydrate' OR 'timolol, ophthalmic' OR 'titol' OR 'propranolol'/exp OR '1 (1 methylethylamino) 3 (1 naphthyloxy) 2 propanol' OR '1 (1 methylethylamino) 3 (1 naphthyloxy) propan 2 ol' OR '1 (2 hydroxy 3 isopropylaminopropoxy) naphthalene' OR '1 (2 propanylamino) 3 (1 naphthyloxy) propan 2 ol' OR '1 (isopropylamino) 3 (1 naphthyloxy) 2 propanol' OR '1 (propan 2 ylamino) 3 (1 naphthyloxy) propan 2 ol' OR '1 isopropylamino 3 (1 naphthoxy) 2 propanol' OR '1 isopropylamino 3 (1 naphthyloxy) propan 2 ol' OR 'acifol' OR 'adrexan' OR 'alperol' OR 'anaprilin' OR 'anapriline' OR 'anaprilinium' OR 'anapryline' OR 'angilol' OR 'angilol la' OR 'apo-propranolol' OR 'apsolol' OR 'arcablock' OR 'arcablock retard' OR 'artensol' OR 'authus' OR 'avlocardyl' OR 'avlocardyl retard' OR 'ay 64043' OR 'ay64043' OR 'becardin' OR 'bedranol' OR 'beprane' OR 'bercolol' OR 'berkolol' OR 'beta neg' OR 'beta tablinen' OR 'beta tablinen retard' OR 'beta timelets' OR 'beta-timelets' OR 'betabloc' OR 'betadipresan' OR 'betaneg' OR 'betaprol' OR 'betares' OR 'betaryl' OR 'blocard' OR 'blocaryl' OR 'cardinol' OR 'cardinol la' OR 'ciplar' OR 'corbeta' OR 'deralin' OR 'dextrolevo propranolol' OR 'dibudinate' OR 'dideral' OR 'dl propanolol hydrochloride' OR 'dl propranolol' OR 'dociton' OR 'dociton retard' OR 'docitone' OR 'durabeton' OR 'duranol' OR 'efektolol' OR 'efektolol retard' OR 'elbrol' OR 'emforal' OR 'farmadral' OR 'farprolol' OR 'frekven' OR 'frina' OR 'hemangeol' OR 'hemangiol' OR 'hopranolol' OR 'ici 45520' OR 'ikopal' OR 'impral' OR 'inderal' OR 'inderal la' OR 'inderal retard' OR 'inderalici' OR 'inderex' OR 'indicardin' OR 'indobloc' OR 'innopran' OR 'innopran xl' OR 'inpanol' OR 'ipran' OR 'l propranolol' OR 'lederpronol' OR 'levo propranolol' OR 'levopropranolol' OR 'm 7030' OR 'm7030' OR 'napriline' OR 'noloten' OR 'nsc 91523' OR 'obsidan' OR 'obsin' OR 'obzidan' OR 'oposim' OR 'phanerol' OR 'prandol' OR 'prano puren' OR 'pranopuren' OR 'prestoral' OR 'prolol' OR 'prolol plus' OR 'pronovan' OR 'propabloc' OR 'propal' OR 'propalong' OR 'propanolol hydrochloride' OR 'propayerst' OR 'propercuten' OR 'prophylux' OR 'propra ratiopharm' OR 'propral' OR 'propranolol' OR 'propranolol hydrochloride' OR 'propranolol hydrochloride intensol' OR 'propranolol isomer' OR 'propranur' OR 'proprasylyt' OR 'proprasylyte' OR 'rec 0551' OR 'rec0551' OR 'reducor' OR 'sagittol' OR 'slow deralin' OR 'stapranolol' OR 'sumial' OR 'tenomal' OR 'tensiflex' OR 'waucoton' OR 'betaxolol'/exp OR '1 (isopropylamino) 3 [4 (cyclopropylmethoxyethyl) phenoxy] 2 propanol' OR '1 [ [4 [2 [ (cyclopropylmethyl) oxy] ethyl] phenyl] oxy] 3 (isopropylamino) isopropanol' OR '1 [ [4 [2 [ (cyclopropylmethyl) oxy] ethyl] phenyl] oxy] 3 [ (1 methylethyl) amino] 2 propanol' OR '1 [ [4 [2 [ (cyclopropylmethyl) oxy] ethyl] phenyl] oxy] 3 [ (1 methylethyl) amino] propan 2 ol' OR '1 [ [4 [2 [ (cyclopropylmethyl) oxy] ethyl] phenyl] oxy] 3 [ (2 propanyl) amino] 2 propanol' OR '1 [ [4 [2 [ (cyclopropylmethyl) oxy] ethyl] phenyl] oxy] 3 [ (propan 2 yl) amino] propan 2 ol' OR '1 [4 (2 cyclopropylmethoxyethyl) phenoxy] 3 isopropylamino 2 propanol' OR '1 [4 (2 cyclopropylmethoxyethyl) phenoxy] 3 isopropylaminoisopropanol' OR '1 [4 (2 cyclopropylmethoxyethyl) phenoxy] 3 isopropylaminopropan 2 ol' OR '1 [4 [2 (cyclopropylmethoxy) ethyl] phenoxy] 3 (propan 2 ylamino) propan 2 ol' OR '1 [4 [2 (cyclopropylmethoxy) ethyl] phenoxy] 3 [ (1 methylethyl) amino] 2 propanol' OR '1 [4 [2 (cyclopropylmethoxy) ethyl] phenoxy] 3 [ (1 methylethyl) amino] propan 2 ol' OR '1 [4 [2 (cyclopropylmethoxy) ethyl] phenoxy] 3 [ (2 propanyl) amino] 2 propanol' OR '1 [4 [2 (cyclopropylmethoxy) ethyl] phenoxy] 3 [ (isopropanyl) amino] isopropanol' OR '1 [4 [2 (cyclopropylmethoxy) ethyl] phenoxy] 3 [ (propan 2 yl) amino] propan 2 ol' OR '1 [4 [2 (cyclopropylmethyloxy) ethyl] phenoxy] 3 [ (1 methylethyl) amino] 2 propanol' OR '1 [4 [2 (cyclopropylmethyloxy) ethyl] phenoxy] 3 [ (1 methylethyl) amino] propan 2 ol' OR '1 [4 [2 (cyclopropylmethyloxy) ethyl] phenoxy] 3 [ (2 propanyl) amino] 2 propanol' OR '1 [4 [2 (cyclopropylmethyloxy) ethyl] phenoxy] 3 [ (isopropanyl) amino] isopropanol' OR '1 [4 [2 (cyclopropylmethyloxy) ethyl] phenoxy] 3 [ (propan 2 yl) amino] propan 2 ol' OR '[4 (2 cyclopropylmethoxyethyl) 3 isopropylaminophenoxy] propan 2 ol' OR 'alcon betoptic' OR 'alo 1401 02' OR 'alo 140102' OR 'alo140102' OR 'betac' OR 'betarun' OR 'betasel' OR 'betaxolol' OR 'betaxolol hydrochloride' OR 'betaxon' OR 'betoptic' OR 'betoptic s' OR 'betoptima' OR 'betoquin' OR 'kerlon' OR 'kerlone' OR 'kerlong' OR 'levobetaxolol' OR 'levobetaxolol hydrochloride' OR 'lokren' OR 'optibet' OR 'optipress' OR 'sl 75212' OR 'tonobexol') AND ('complete response'/exp OR 'partial response'/exp OR 'common terminology criteria for adverse events'/exp OR 'ctcae dictionary' OR 'common terminology adverse events' OR 'common terminology criteria adverse event' OR 'common terminology criteria adverse events' OR 'common terminology criteria for adverse event' OR 'common terminology criteria for adverse events' OR 'common terminology criteria for adverse events (ctcae)' OR 'common terminology criteria of adverse events' OR 'common terminology criteria of adverse event (nci-ctcae)' OR 'common terminology for adverse events criteria' OR 'common terminology of adverse events' OR 'common toxicity criteria adverse event' OR 'common toxicity criteria adverse events' OR 'common toxicity criteria for adverse event' OR 'common toxicity criteria for adverse events' OR 'common toxicity criteria of adverse event' OR 'common toxicity criteria of adverse events' OR 'common toxicity criteria of adverse events (ctc)' OR 'common terminology for adverse event' OR 'common toxicity criteria for the adverse events' OR 'pain assessment'/exp OR 'pain assessment' OR 'pain scale' OR 'quality of life'/exp OR 'hrql' OR 'health related quality of life' OR 'life quality' OR 'quality of life') |
| Cochrane |
| ((([mh "erbb receptors"] OR (erbb AND receptors) OR "erbb receptors" OR (epidermal AND growth AND factor AND receptor) OR "epidermal growth factor receptor") AND ([mh /"antagonists and inhibitors"] OR (antagonists AND inhibitors) OR "antagonists and inhibitors" OR inhibitors OR inhibitor OR "inhibitor s")) OR ([mh gefitinib] OR gefitinib OR "gefitinib s" OR ([mh "erlotinib hydrochloride"] OR (erlotinib AND hydrochloride) OR "erlotinib hydrochloride" OR erlotinib OR "erlotinib s") OR ([mh afatinib] OR afatinib) OR (osimertinib:kw OR osimertinib))) AND (("adrenergic beta antagonists" OR [mh "adrenergic beta antagonists"] OR (adrenergic AND "beta antagonists") OR "adrenergic beta antagonists" OR (beta AND blocker) OR "beta blocker" OR ([mh propranolol] OR propranolol OR "propranolol s" OR propranolols) OR ([mh timolol] OR timolol) OR ([mh betaxolol] OR betaxolol)) AND ([mh "granuloma, pyogenic"] OR (granuloma AND pyogenic) OR "pyogenic granuloma" OR (pyogenic AND granuloma) OR ([mh paronychia] OR paronychia OR paronychias))) |
| **SCOPUS** |
| ( paronychia OR "pseudopyogenic granulomas" OR "pyogenic granuloma-like" ) AND ( tki OR "tyrosine kinase inhibitor" ) AND ( "beta blocker" OR "Timolol" OR "propranolol" OR "betaxolol" ) |

**Table S3. PICO criteria**

| Population | Epidermal growth factor receptor inhibitor -induced Paronychia or pyogenic granuloma-like lesions |
| --- | --- |
| Intervention | Topical Beta Blocking Agent  (such as Timolol OR betaxolol OR propranolol) |
| Comparison | Baseline status |
| Outcome | Primary outcome   - Overall response rate - Complete response rate (person or lesion) - Partial response rate (person or lesion)   Secondary outcome   - CTCAE scores - Pain score - Adverse event |

**Table S4. Assessment of risk of bias**

Risk of bias assessment fore case report or case series developed by Murad et al

| Domains | Leading explanatory questions |
| --- | --- |
| Selection | 1. Does the patient(s) represent(s) the whole experience of the investigator (centre) or is the selection method unclear to the extent that other patients with similar presentation may not have been reported? |
| Ascertainment | 2. Was the exposure adequately ascertained? 3. Was the outcome adequately ascertained? |
| Causality | 4. Were other alternative causes that may explain the observation ruled out? 5. Was there a challenge/rechallenge phenomenon? 6. Was there a dose–response effect? 7. Was follow-up long enough for outcomes to occur? |
| Reporting | 8. Is the case(s) described with sufficient details to allow other investigators to replicate the research or to allow practitioners make inferences related to their own practice? |

| Study | Selection | Ascertainment | Causality | Reporting | Final Score |
| --- | --- | --- | --- | --- | --- |
| Cubiro et al. 2018 | Low | Low | Low | Low | Low |
| Sollena et al. 2019 | Low | Low | Low | Low | Low |
| Sibaud et al. 2019 | Some Concern | Low | Low | Low | Low |
| Olamiju et al. 2020 | Low | Low | Some Concern | Low | Low |
| Yen et al. 2020 | Low | Low | Low | Low | Low |
| Liu et al. 2022 | Low | Low | Low | Low | Low |

Newcastle‐Ottawa scale score

|  | Selection | | | | Comparability | Outcome | | | |  | |
| --- | --- | --- | --- | --- | --- | --- | --- | --- | --- | --- | --- |
| Study | Representativeness  of exposed cohort | Selection of  the control | Ascertainment of exposure | Outcome of  interest was not  present at start  of study | Control of important and additional factors | Assessment  of outcome | Follow-up  long enough for outcomes to occur | Adequacy of  follow up of  cohorts | Final Score | |  |
| Cubiro 2018 | V | - | V | V | - | V | V | V | 6 | |  |
| Sollena 2019 | V | - | V | V | - | V | V | V | 6 | |  |
| Sibaud 2019 | V | - | V | V | - | V | V | - | 5 | |  |
| Olamiju 2020 | V | - | V | V | - | V | V | - | 5 | |  |
| Yen 2020 | V | - | V | V | - | V | V | V | 6 | |  |
| Liu 2022 | V | - | V | V | - | V | V | - | 5 | |  |

**Table S5. Meta-regression results for overall response and complete response**

| **Outcome/Predictor** | **No. of studies** | **Coefficient** | **CI** | **P_value** |
| --- | --- | --- | --- | --- |
| **Complete response** | | | | |
| Publication year | 6 | -0.109 | -0.291 - 0.074 | 0.242 |
| Gender, female % | 6 | -1.700 | -3.603 - 0.204 | 0.080 |
| Onset, month | 6 | -0.024 | -0.167 - 0.12 | 0.747 |
| TKI use, % | 6 | -0.581 | -1.259 - 0.097 | 0.093 |
| Afatinib use, % | 6 | -0.399 | -1.311 - 0.512 | 0.390 |
| Lesion amounts | 4 | 0.000 | -0.006 - 0.012 | 0.993 |
| Lesion only on hands, % | 5 | 0.503 | -0.891 - 1.896 | 0.480 |
| Lesion only on feet, % | 5 | 1.764 | -0.876 - 4.405 | 0.190 |
| Lesion on both, % | 5 | -0.591 | -1.859 - 0.676 | 0.361 |
| **Overall response** | | | | |
| Publication year | 6 | -0.003 | -0.069 - 0.063 | 0.927 |
| Gender, female % | 6 | -0.162 | -0.96 - 0.635 | 0.690 |
| Onset, month | 6 | -0.011 | -0.054 - 0.032 | 0.622 |
| TKI use, % | 6 | -0.041 | -0.318 - 0.236 | 0.772 |
| Afatinib use, % | 6 | 0.073 | -0.220 - 0.367 | 0.624 |
| Lesion amounts | 4 | 0.000 | -0.001 - 0.001 | 0.697 |
| Lesion only on hands, % | 5 | 0.168 | -0.332 - 0.668 | 0.510 |
| Lesion only on feet, % | 5 | -0.049 | -1.125 - 1.027 | 0.929 |
| Lesion on both, % | 5 | -0.102 | -0.558 - 0.355 | 0.662 |

TKI, tyrosine kinase inhibitor; CI, confidence interval; No., number

**Supplement Table S6. Summary of result and certainty of evidence**

| **Number of studies** | **Study design** | **Number of Participants** | **Pooled Response Rate (95% CI)** | **Certainty of Evidence (GRADE)** | **Comments** |
| --- | --- | --- | --- | --- | --- |
| **Overall Response rate** | | | | | |
| 6 | observational studies | 96 | **94%**  (81-100%) | **⊕◯◯◯**  **VERY LOW**ᵃ | Topical beta-blockers showed high overall response rate |
| **Complete Response rate** | | | | | |
| 6 | observational studies | 96 | **34%**  (15-57%) | **⊕◯◯◯**  **VERY LOWᵃ** | Topical beta-blockers showed moderate complete response rate |

**ᵃ** The initial low certainty, inherent to single-arm observational designs, was further downgraded due to serious inconsistency (indicated by high heterogeneity) and serious imprecision (attributable to small sample sizes)

**Supplement S1. Forest plot analysis of study-level subgroup analysis of regimens for (A) overall response and (B) complete response**

**
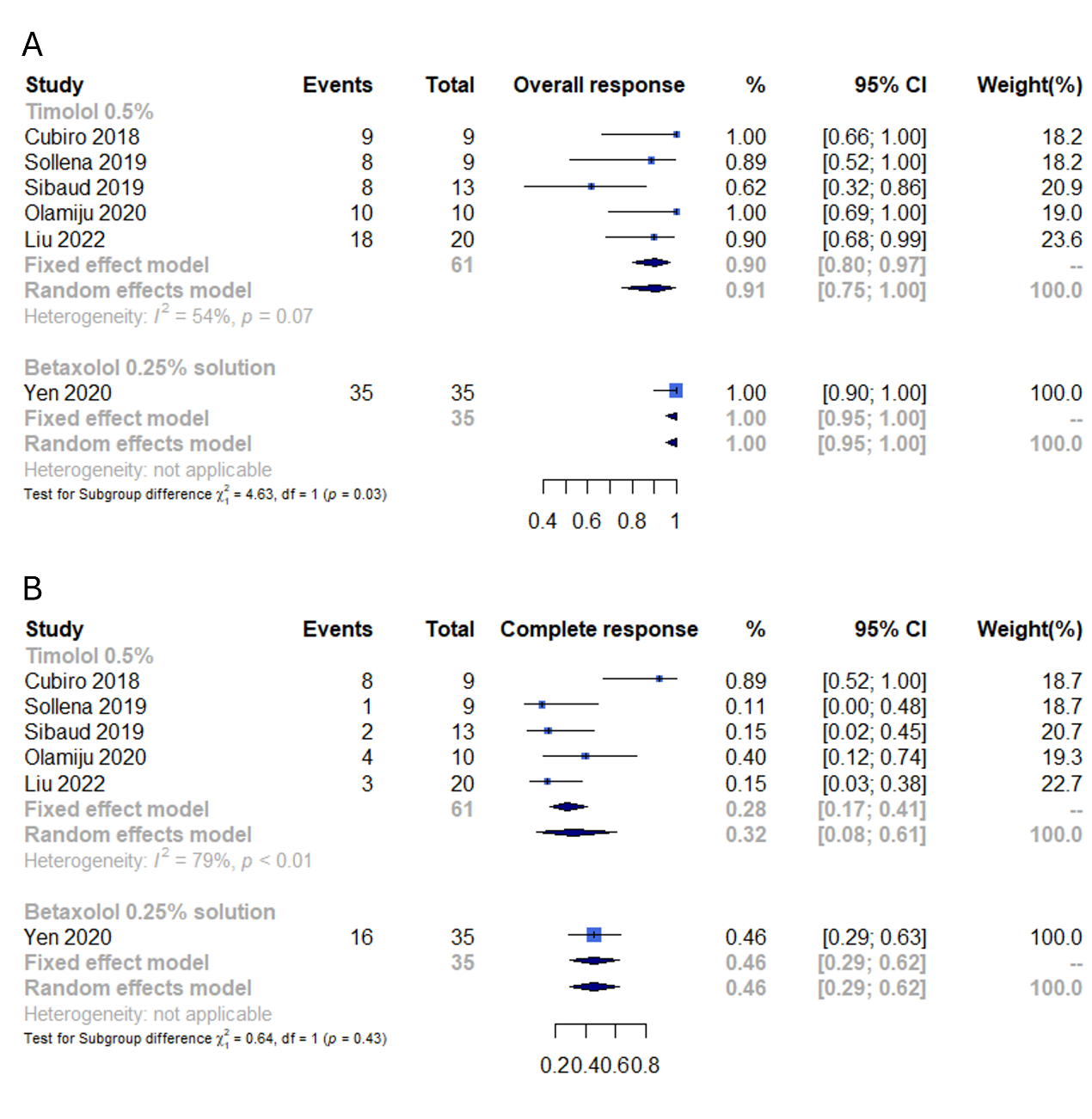
**

**Fig S2. Forest plot analysis of study-level subgroup analysis of geographic differences for (A) overall response and (B) complete response
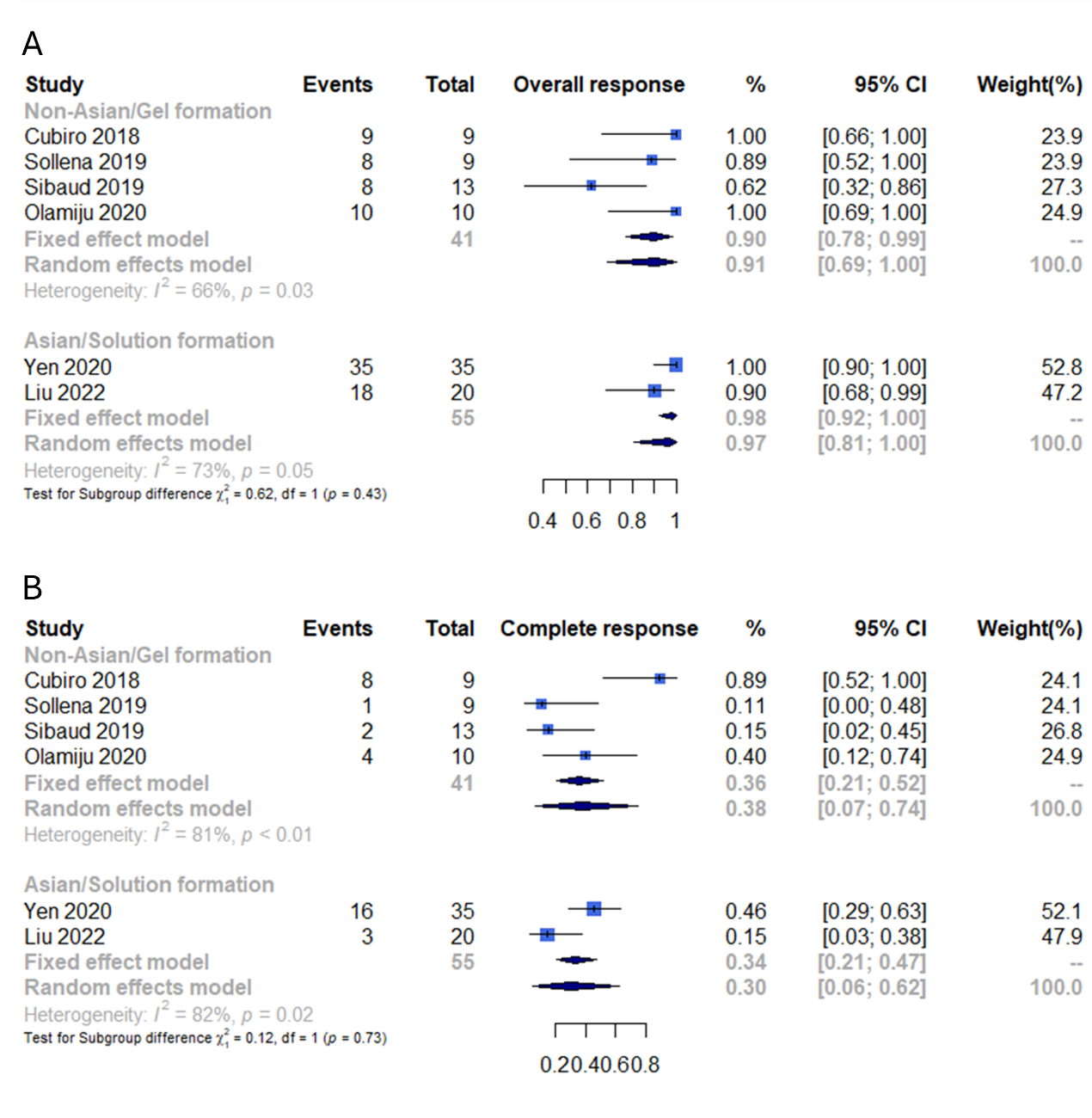
**

**Fig S3: Sensitivity test of (A) overall response and (B) complete response excluding Cubiro 2018.
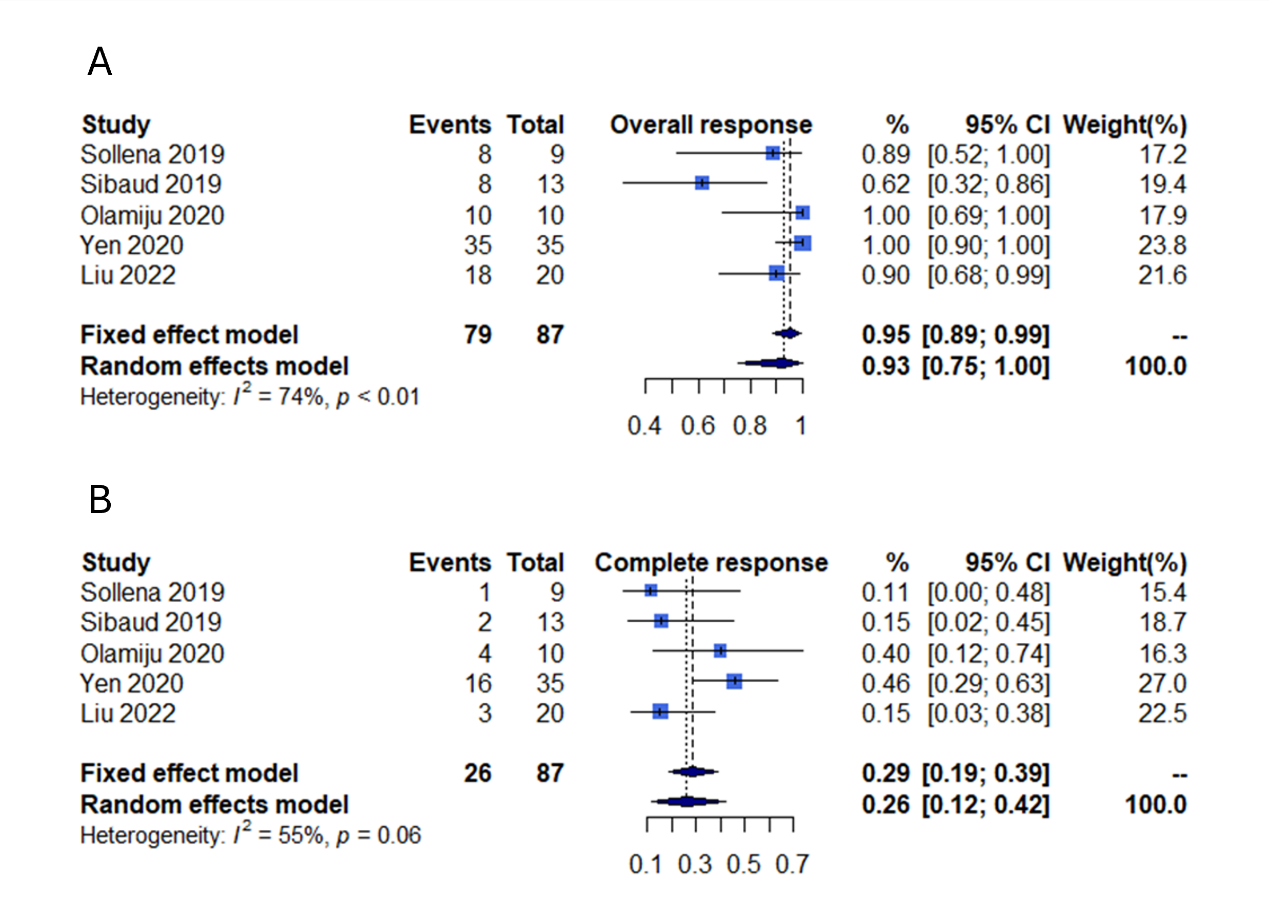
**

**Fig S4. Funnel plots
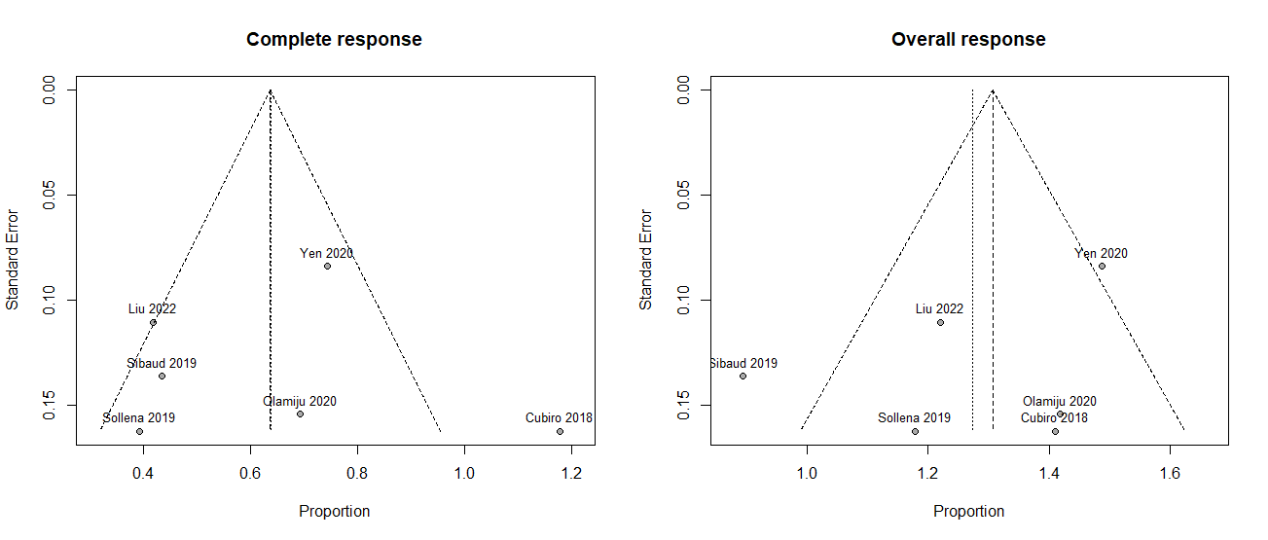
**

**Fig S5
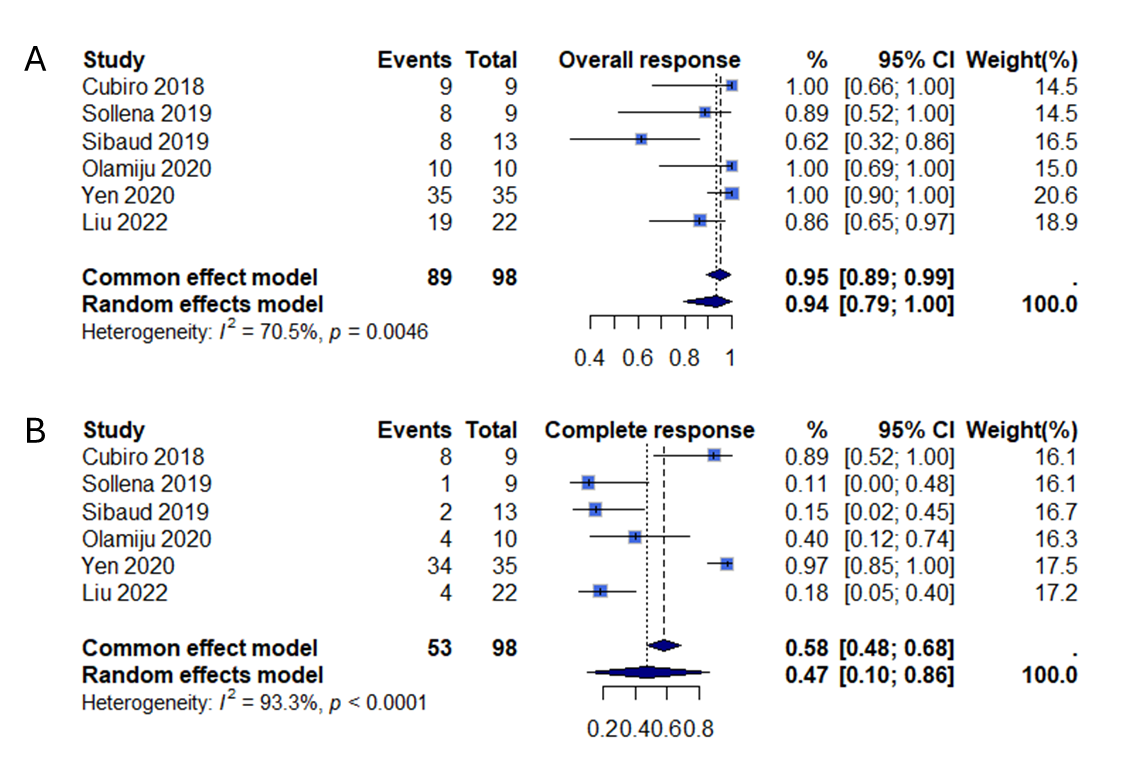
**
